# Identifying B-cell epitopes using AlphaFold2 predicted structures and pretrained language model

**DOI:** 10.1093/bioinformatics/btad187

**Published:** 2023-04-11

**Authors:** Yuansong Zeng, Zhuoyi Wei, Qianmu Yuan, Sheng Chen, Weijiang Yu, Yutong Lu, Jianzhao Gao, Yuedong Yang

**Affiliations:** School of Computer Science and Engineering, Sun Yat-sen University, Guangzhou 510000, China; School of Computer Science and Engineering, Sun Yat-sen University, Guangzhou 510000, China; School of Computer Science and Engineering, Sun Yat-sen University, Guangzhou 510000, China; School of Computer Science and Engineering, Sun Yat-sen University, Guangzhou 510000, China; School of Computer Science and Engineering, Sun Yat-sen University, Guangzhou 510000, China; School of Computer Science and Engineering, Sun Yat-sen University, Guangzhou 510000, China; School of Mathematical Sciences and LPMC, Nankai University, Tianjin 300072, China; School of Computer Science and Engineering, Sun Yat-sen University, Guangzhou 510000, China; Key Laboratory of Machine Intelligence and Advanced Computing (MOE), Sun Yat-sen University, Guangzhou 510000, China

## Abstract

**Motivation:**

Identifying the B-cell epitopes is an essential step for guiding rational vaccine development and immunotherapies. Since experimental approaches are expensive and time-consuming, many computational methods have been designed to assist B-cell epitope prediction. However, existing sequence-based methods have limited performance since they only use contextual features of the sequential neighbors while neglecting structural information.

**Results:**

Based on the recent breakthrough of AlphaFold2 in protein structure prediction, we propose GraphBepi, a novel graph-based model for accurate B-cell epitope prediction. For one protein, the predicted structure from AlphaFold2 is used to construct the protein graph, where the nodes/residues are encoded by ESM-2 learning representations. The graph is input into the edge-enhanced deep graph neural network (EGNN) to capture the spatial information in the predicted 3D structures. In parallel, a bidirectional long short-term memory neural networks (BiLSTM) are employed to capture long-range dependencies in the sequence. The learned low-dimensional representations by EGNN and BiLSTM are then combined into a multilayer perceptron for predicting B-cell epitopes. Through comprehensive tests on the curated epitope dataset, GraphBepi was shown to outperform the state-of-the-art methods by more than 5.5% and 44.0% in terms of AUC and AUPR, respectively. A web server is freely available at http://bio-web1.nscc-gz.cn/app/graphbepi.

**Availability and implementation:**

The datasets, pre-computed features, source codes, and the trained model are available at https://github.com/biomed-AI/GraphBepi.

## 1 Introduction

B-cells are a crucial element of the immune system to provide immunological protection against harmful molecules or infectious pathogens by producing antibodies that bind with antigens (Delves et al. 2016). The specific region of an antigen binding to an antibody is known as antigenic determinant or an epitope (Paul 2012). The category of B-cell epitopes (BCEs) is widely classified into two groups: linear and conformational epitopes (Alghamdi et al. 2022). Linear epitopes include continuous amino acid residues, whereas conformational epitopes are shaped by a 3D conformation that folds the protein to bind through the interaction of discontinuous amino acid residues. Previous studies show that more than 90% of BCEs are conformational while 10% are linear epitopes (Barlow et al. 1986).

Reliable tools for the identification of BCEs are important in biotechnological and clinical applications (e.g. therapeutic antibody development and vaccine design, as well as in the overall understanding of immune mechanisms) (Gomara and Haro 2007). X-ray crystallography and nuclear magnetic resonance techniques are trustable approaches for identifying BCEs (Mayer and Meyer 2001). Nevertheless, these traditional experimental approaches are expensive and time-consuming (Kavitha et al. 2013). The silico prediction tools can mitigate the identification workload by predicting epitope regions. For example, Jespersen et al. (2017) propose the commonly used tool BepiPred-2.0, which employs a random forest model to train annotated epitopes from antibody-antigen protein structures and then uses the trained model to predict newly generated antigen sequences. Afterward, with the increase in biological data, a few deep learning methods are implemented for accurately predicting BCEs. EpiDope (Collatz et al. 2021) uses a deep neural network to identify BCEs on individual protein sequences, which extracts context-aware representations for every residue in the sequence via applying the feature vector that has a length of 1000. In this way, EpiDope exceeds baseline methods in identifying BCEs. Although the above methods obtain good performance in identifying linear epitopes, they have difficulty in identifying conformational epitopes consisting of amino acid fragments that are far apart in the protein sequence but are brought together by the conformational folding of the polypeptide chain.

To solve these problems, several structure-based methods have been designed by considering spatial information. DiscoTope (Haste Andersen et al. 2006) is the first approach that focuses on discontinuous epitopes by considering spatial information, amino acid statistics, and surface accessibility. DiscoTope-2.0 (Kringelum et al. 2012) is the improved version of DiscoTope by adding half-sphere exposure and propensity scores as a surface measure. Nonetheless, DiscoTope-2.0 does not take into account glycosylation which may significantly affect epitopes. SEPPA 3.0 (Zhou et al. 2019) investigates the impact of glycosylation in the antigen surface patches, showing that antibodies may tend to attach in N-glycosylation sites. ElliPro (Ponomarenko et al. 2008) characterizes antigenic proteins by approximating them as ellipsoids and then calculates the protrusion index of the residues to cluster them. Epitope3D (da Silva et al. 2022) is a new scalable machine learning approach for predicting conformational epitopes by using graph-based structural signatures. So far, structure-based tools have achieved decent performance, most of the time, better than sequence-based tools (Singh et al. 2013, da Silva et al. 2022). However, since experimentally decided structural information is usually not available, BCEs prediction must in numerous cases be conducted via sequences alone.

With the great advances in deep learning technologies, protein structure prediction is undergoing a breakthrough. For example, AlphaFold2 (Jumper et al. 2021) is a complicated deep-learning model for predicting protein structures, which has integrated a lot of biological and physical knowledge. In the 14th Critical Assessment of Protein Structure Prediction, AlphaFold2 has shown the ability in predicting the structure of the protein with atomic accuracy and demonstrated accuracy competitive with experiments on a large number of cases. On the other hand, unsupervised pre-training using language models has led to breakthrough improvements in natural language processing. Recently, these techniques have been employed in protein sequence representation learning and have shown very promising results in many prediction tasks such as tertiary contacts, mutational effects, and secondary structure (Elnaggar et al. 2020, Yuan et al. 2022). These breakthroughs inspire us to design an accurate BCE predictor by using the predicted protein structures and the pretrained language model.

In this study, we propose GraphBepi, a novel graph-based model for accurate epitope prediction. GraphBepi first generates the effective information sequence representations and protein structures from antigen sequences by the pretrained language model and AlphaFold2, respectively. GraphBepi then applies the edge-enhanced deep graph neural network (EGNN) (Gong and Cheng 2019) to capture the predicted protein structural information and leverages the bidirectional long short-term memory neural networks (BiLSTM) (Hochreiter and Schmidhuber 1997) to capture long-range dependencies from sequences. The low-dimensional representation learned from EGNN and BiLSTM is then combined to predict BCEs. Through comprehensive tests on the curated epitope dataset, GraphBepi was shown to outperform the state-of-the-art methods.

## 2 Materials and methods

### 2.1 Dataset

To train and evaluate our model, we took a similar strategy as the study (da Silva et al. 2022) to build a large epitope dataset. Specifically, we first fetched all biological assemblies with a value of resolution greater than or equal to 3 Å from the Protein Data Bank deposited before 09 May 2022 (Berman et al. 2000). Next, the ANARCI (Dunbar et al. 2016) tool was used to identify antibody-antigen complexes and retain antigen chains with lengths of 25–1024. We labeled epitope residues in the antigen molecule depending on a cutoff distance standard. For example, an antigen residue that has at least one heavy atom at a distance of <4 Å to a residue of antibody will be treated as the epitope residue. We removed the antigen chain if it contained epitopes <5. Next, we used MMseqs2 (Steinegger and Söding 2017) to cluster antigen sequences, and any antigen sequence belonging to the same cluster was aligned to the cluster representative defined by MMseqs2 through tool blastp (Johnson et al. 2008). Each clustering representative sequence was then modified as follows: if an epitope was labeled in the clustering representative, it would be kept. If one epitope was found in any antigen sequence of the aligned sequences, it would be transposed to the clustering representative sequence and marked as an epitope. This process was done at 95% sequence identity, resulting in the size of the dataset with 783 antigen sequences. We further conducted redundancy reduction via MMseqs2 at 70% sequence identity, resulting in generating a nonredundant dataset of 633 antigen sequences. Finally, the antigen sequences deposited after 04 January 2021 were used as the independent test data (56 antigen sequences, consisting of 1393 binding residues, and 14 150 nonbinding residues, with 8.96% of epitope residues), and the rest of the antigen sequences were used as the training data (577 antigen sequences, consisting of 15 981 binding residues, and 119 869 nonbinding residues, with 11.76% of epitope residues).

### 2.2 Protein representation

#### 2.2.1 Language model representation

The latest language model esm2_t36_3B_UR50D (Lin et al. 2022) (denoted as ESM-2) is used for extracting features from each antigen sequence, which is an updated version of esm_msa1b_t12_100M_UR50S (denoted as ESM). The architecture of ESM and ESM-2 is based on the transformer model, and both of them are pretrained on UniRef50 (Suzek et al. 2007) through the masked language modeling objective (Devlin et al. 2018) in an unsupervised manner. We leverage the ESM-2 to extract sequence representation for each residue, which generates a 2560D feature vector for per-residue. We introduce ESM-2 in the Supplementary Note S1. We have also tested another similar protein language model ProtT5-XL-U50 (Elnaggar et al. 2020) (denoted as ProtTrans), which is first trained on BFD (Steinegger et al. 2019) and then fine-tuned on the UniRef50.

#### 2.2.2 Evolutionary information

Evolutionarily conserved residues probably include motifs correlated to crucial properties of the protein. For investigating the importance of evolutionary features, we test the widely used evolutionary features HMM profile and position-specific scoring matrix (PSSM). The HMM profiles are produced by running the tool HHblits (Remmert et al. 2012) against UniClust30 (Mirdita et al. 2017) using the default setting. The PSSM is produced by conducting the tool PSI-BLAST (Altschul et al. 1997) to seek the candidate sequence against UniRef90 (Suzek et al. 2007) using an E-value of 0.001 and three iterations. Each residue is embedded into a 20D feature vector through PSSM and HMM, respectively. We detail PSSM and HMM in Supplementary Note S2.

#### 2.2.3 Predicted protein structures

To take account of spatial information for each residue, we apply the predictive tool AlphaFold2 to predict protein structure. Specifically, we follow the tutorial at https://github.com/deepmind/alphafold to deploy AlphaFold2 on the Tianhe-2 supercomputer and then predict the protein structures. AlphaFold2 is detailed in Supplementary Note S3. We have also investigated another similar protein structural prediction model, esmfold_v1 (Lin et al. 2022) (denoted as ESMFold), which is a full end-to-end single sequence structure predictor. We download the pretrained ESMFold and then directly apply it to predict protein structures.

#### 2.2.4 Structural properties

We apply the DSSP program (Kabsch and Sander 1983) to extract three kinds of structural properties from the AlphaFold2 predicted protein structures: (1) the profile of the 9D one-hot secondary structure, where the main eight dimensions indicate the states of the eight secondary structures and the final dimension indicates the unknown secondary structure. (2) Relative solvent accessibility (RSA) is obtained by normalizing the solvent accessible surface area (ASA) through the maximal possible ASA of the corresponding amino acid type. (3) Peptide backbone torsion angles PSI and PHI are transferred into a 4D feature vector by cosine and sine. In summary, these structural feature vectors with 13 dimensions are called DSSP in this manuscript.

### 2.3 The architecture of GraphBepi

**Figure 1.**
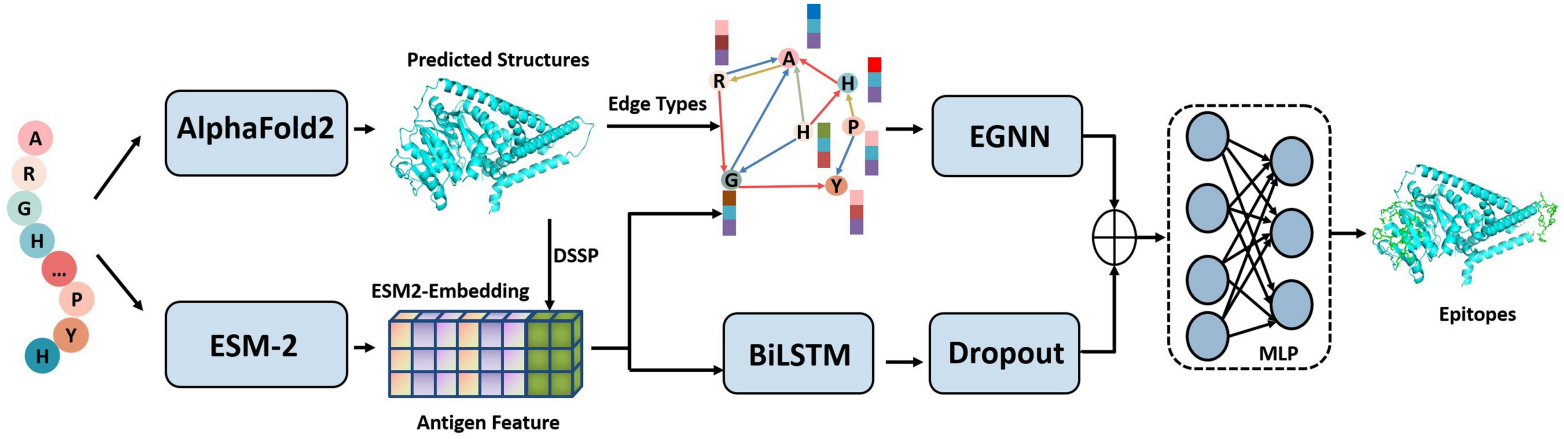
The framework of the GraphBepi model. The input antigen sequence is respectively fed into the pretrained language model and AlphaFold2 to generate the ESM2-embedding and protein structures. The relational graph of residues and DSSP are extracted from the predicted protein structures. The ESM2-embedding and DSSP are then fed into the BiLSTM module to learn the effective representation by capturing long-range dependencies of the residues. They are also concatenated to form feature vectors of residues in the relational graph, which is then fed into the EGNN to learn the structural information. Finally, the output of the EGNN and BiLSTM modules is concatenated to predict BCEs through an MLP.

This study proposes a novel method GraphBepi for improving BCEs prediction by considering spatial information. As shown in Fig. 1, the antigen sequence is fed into the pretrained language model and AlphaFold2 to generate the sequence embedding and protein structures, respectively. The relational graph of residues and DSSP are then extracted from protein structures. The sequence embedding and DSSP are then fed into the BiLSTM module to learn the effective representation by capturing long-range dependencies from sequences. They are also concatenated to form feature vectors of residues in the relational graph and are then fed into the EGNN to learn the structural information. Finally, the output of the EGNN and BiLSTM modules is concatenated to predict BCEs through a multilayer perceptron (MLP).

#### 2.3.1 The bidirectional LSTM module

The long short-term memory (LSTM) is a classic algorithm for capturing long-range dependencies, which is widely used in protein sequence encoding. The BiLSTM model appends one more LSTM layer and reverses the direction of information. Namely, it represents that the input sequence passes backward in the newly added LSTM layer. In this study, we use a bidirectional LSTM to process antigen sequences since they do not have a specific direction. We indicate the output of the BiLSTM using the term H for simplicity. The BiLSTM is employed for learning the DSSP structural properties obtained from predicted structures and the sequence embeddings obtained from ESM-2, respectively. We introduce the BiLSTM algorithm carefully in Supplementary Note S4.

#### 2.3.2 Graph construction

We build the residue-level relational graph G=(N,ε, R) for the structure of an antigen following Zhang et al. (2022), where N and ε  mean the set of residues (nodes) and edges, respectively, and R is the set of edge types. The term (i,j,r) is used for indicating the edge from node i to node j with edge type r. We add three types of directed edges into the graph including K-nearest neighbor edges, radius edges, and sequential edges. They are generated as follows: (1) sequential edges: residues i and j will be connected by an edge if ${\left|j-i\right|<d}_{\mathrm{seq}}$, where |j-i| represents the sequential distance between residues i and j, and $d_{\mathrm{seq}}$ is a predefined threshold. Then, the d=j-i is used as the edge type between residue i and *j*. Hence, there are 2$d_{\mathrm{seq}}-$1 types of sequential edges. (2) Radius edges: the radius edges between two residues are also added when the spatial distance between them is less than a threshold $d_{\mathrm{radius}}$. (3) K-nearest neighbor edges: KNN edge types are added by computing the K-nearest neighbors based on the Euclidean distance. In this study, the sequential distance threshold $d_{\mathrm{seq}}$ and the radius are set to 3 and 10, respectively. The number of residue neighbor *k* is set to 10. Finally, the edge types consist of radius edges, KNN edges, and sequential edges, resulting in 2$d_{\mathrm{seq}}$+1 = 7 different types of edges. We further introduce these edge types in Supplementary Note S5.

#### 2.3.3 EGNN module

We apply the EGNN (Gong and Cheng 2019) framework to capture spatial information by adequately integrating node (residue) features and multiple-dimensional edge features. Given a residue graph with *N* residues, we first let X be an N×F matrix representing the residue features of the entire graph, where *F* is the dimension of the residue vector. The edge features are represented by an N×N×P tensor, where P is the dimension of the edge feature. Therefore, $E_{ij}$ indicates the P-dimensional feature of the edge that connects residue i and residue j. The term $E_{\mathrm{ijp}}\;$indicates the pth channel for the edge feature in $E_{ij}.\;$Concretely, the feature vector $X_{i}^{l}\;$of residue i at the l layer will be summed from feature vectors of the neighboring nodes by simultaneously integrating the edge features. The aggregation operation is represented as follows: where σ is the activation function. α is the attention operation, which is guided by edge features of the edge connecting two residues; $\alpha_{.p}$ represents the p channel matrix slice of the N×N×P tensor. Specifically, we treat multiple-dimensional edge features as multi-channel signals and then perform an individual attention for each channel. These results from each channel are then combined through the concatenation operation ||. For an individual channel of edge features, we conduct the attention function as follows: where $f^{l}$ is the attention function implemented by a linear function: where L is the LeakyReLU function. The term | | is the concatenation operation. In Equation (1), g is the transformation function mapping the residue features from the input to the output space, which can be formulated as follows: where $W^{l}$ is a parameter matrix. Finally, the attention coefficients will be treated as the new edge features for the following layer. By this mean, EGNN efficiently integrates the edge and node features.


(1) $$X^{l}=\sigma \left[{\left|\right|}_{p=1}^{P}\left(\alpha_{.p}^{l}\left(X^{l-1},E_{.p}^{l-1}\right)g^{l}\left(X^{l-1}\right)\right)\right]\;$$

(2) $$\alpha_{\mathrm{ijp}}^{l}=f^{l}\left(X_{i}^{l-1},X_{j}^{l-1}\right)E_{\mathrm{ijp}}^{l-1}\;$$

(3) $$f^{l}\left(X_{i.}^{l-1},X_{j.}^{l-1}\right)=\exp\{L(\alpha^{T}[WX_{i.}^{l-1}||WX_{j.}^{l-1}])\}\;$$

(4) $$g^{l}(X^{l-1})=W^{l}X^{l-1}\;$$

#### 2.3.4 Multilayer perceptron

We combine the output of the EGNN module and the BiLSTM module via concatenation operation, and then feed them into the MLP to identify the BCE binding probabilities as follows: where $X_{g}^{\left(L\right)}\in R^{N\times 512},{\;H}_{\mathrm{Dssp}\;}^{\left(L\right)}\in R^{N\times 256},\;\mathrm{and}\;H_{\mathrm{ESM}2-\mathrm{seq}}^{\left(L\right)}\in R^{N\times 256}$ are the output of the last layer of EGNN module and the BiLSTM module, respectively. $Y\in R^{N\times 1}$ is the predictive result of N amino acid residues.


(5) $$Y=\mathrm{Sigmoid}\;\left(\left(X_{g}^{\left(L\right)\;}\left\|\;H_{\mathrm{Dssp}}^{\left(L\right)}\;\right\|\;H_{\mathrm{ESM}2-\mathrm{seq}}^{\left(L\right)}\right)W+b\right)$$

### 2.4 Evaluation metrics and implementation details

Similar to the previous study (Yuan et al. 2021), six commonly used metrics are employed for evaluating prediction performance. They are recall (Rec), precision (Pre), Matthews correlation coefficient (MCC), F1-score (F1), area under the precision-recall curve (AUPR), and area under the receiver operating characteristic curve (AUC): where TN and TP are true negatives and true positives, meaning the number of nonbinding and binding residues predicted correctly, respectively. FN and FP are false negatives and false positives, representing the number of incorrectly identified nonbinding and binding residues, respectively. AUPR and AUR are computed without thresholds, therefore revealing the overall performance of the method. The rest metrics are computed through a predefined threshold to transfer the predicted scores to binary predictions, where the threshold is decided by maximizing the F1 score.


(6) $$\mathrm{Pre}=\frac{\mathrm{TP}}{\mathrm{TP}+\mathrm{FP}}$$

(7) $$\mathrm{Rec}=\frac{\mathrm{TP}}{\mathrm{TP}+\mathrm{FN}}\;$$

(8) $$F1=2\times \frac{\mathrm{Precision}\times \mathrm{Recall}}{\mathrm{Precision}+\mathrm{Recall}}\;$$

(9) $$\mathrm{MCC}=\frac{\mathrm{TP}\times \mathrm{TN}-\mathrm{FN}\times \mathrm{FP}}{\sqrt{\left(\mathrm{TP}+\mathrm{FP}\right)\times \left(\mathrm{TP}+\mathrm{FN}\right)\times \left(\mathrm{TN}+\mathrm{FP}\right)\times \left(\mathrm{TN}+\mathrm{FN}\right)}}\;$$

All experimental results were conducted on an Nvidia GeForce RTX 3090 GPU. We introduce the implementation details in the Supplementary Note S6.

## 3 Results

### 3.1 Performance on the 10-fold CV and independent test

The GraphBepi method was evaluated according to AUC, AUPR, F1, and MCC using the 10-fold CV on the training set together with the independent test. Concretely, the GraphBepi model achieved AUC values of 0.723 and 0.751, AUPR values of 0.245 and 0.261, F1 values of 0.320 and 0.310, and MCC values of 0.212, and 0.232 on the 10-fold CV and the independent test, respectively (Supplementary Table S1). The consistent performance on the CV and independent test demonstrated the robustness of our model. For further investigating the advantages of antigen geometric information and the EGNN, we compared GraphBepi with a baseline model transformer consisting of two-layer networks. The transformer was used as a baseline to test the impact of the structural information for epitope prediction, which was fed the same features as GraphBepi. As shown in Supplementary Table S2, GraphBepi consistently outperformed the baseline model transformer with 4.6%, 6.4%, 5.1%, and 5.6% higher values in terms of AUC, AUPR, F1, and MCC, respectively. The improved performance of our method over the transformer may result from its ability in capturing spatial information, because the EGNN helped GraphBepi pay attention to the spatially adjacent residues, which learned the remote residues connected in the whole graph by efficiently integrating different edge features and residue features. As shown in Supplementary Table S2, the removal of edge features caused a decrease of 2.9% in terms of AUPR on the independent test.

**Figure 2.**
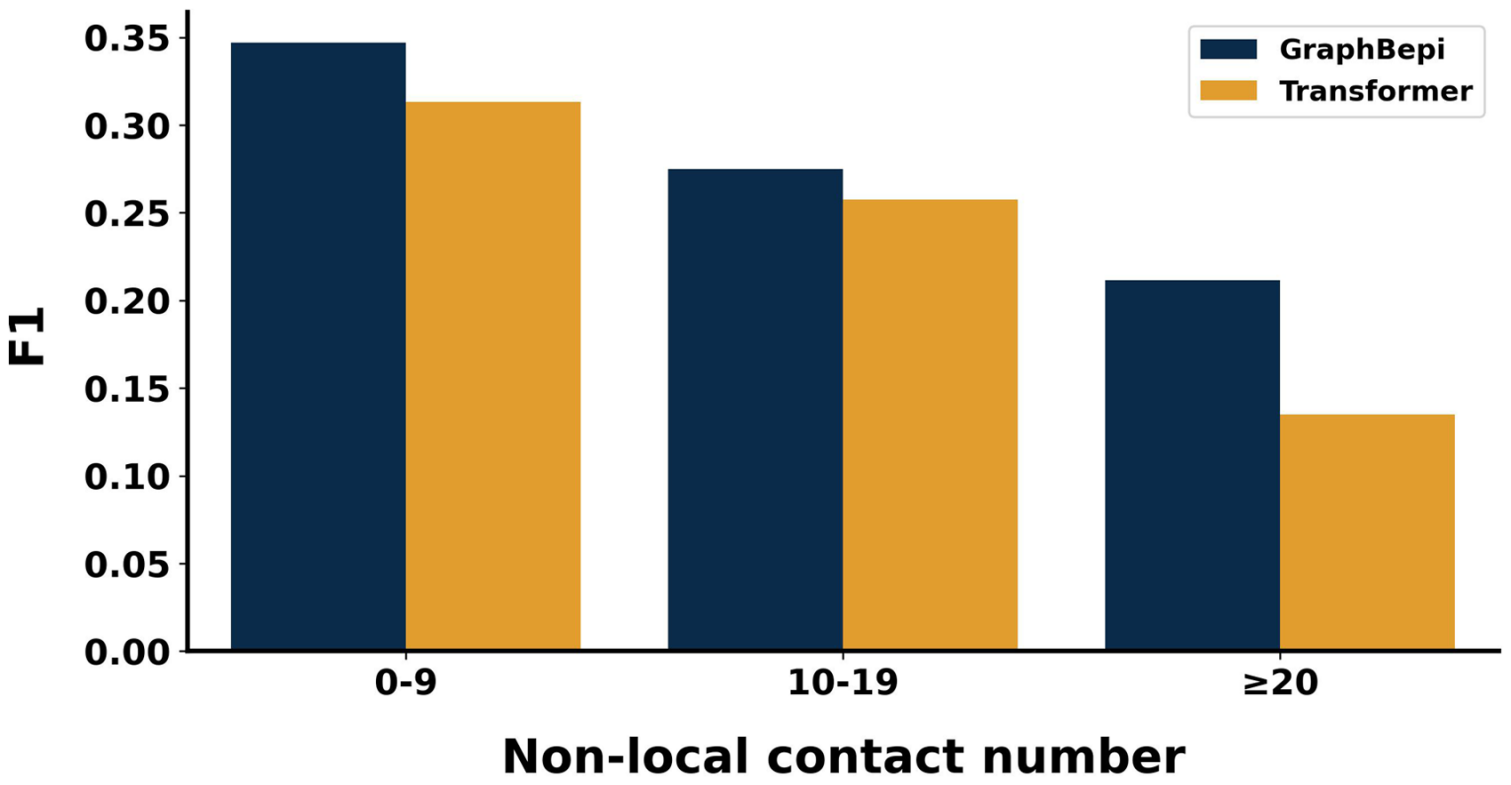
The F1 values of GraphBepi and transformer on amino acids with the different numbers of nonlocal contacts.

We further evaluated the performance of our method and transformer on amino acids with different numbers of nonlocal contacts. Two residues are considered as nonlocal contacts if they are separated with greater than 20 residues in sequence and their Cα distance is <12 Å. Figure 2 shows that our method consistently outperformed the transformer on the independent test data. Concretely, the performance of our method outperformed the transformer by 11% in terms of F1 when the amino acids had 0–9 nonlocal contacts, and the performance gap expanded to 52% when the number of nonlocal contacts were larger than 20. Similar trends could be found when measured by other metrics (Supplementary Fig. S1). The results demonstrated that GraphBepi efficiently captured the spatial information of antigen structures.

### 3.2 Representation from pretrained language models is informative for predicting BCEs

To evaluate the effects of ESM-2 on GraphBepi, we compared ESM-2 with the broadly used evolutionary features, and another two language models (ESM and ProtTrans). As shown in Table 1, on the independent test, the model only using ESM-2 features gained an average AUC and AUPR of 0.736 and 0.240, respectively. By comparison, the uses of only EVO (evolutionary profiles of HMM and PSSM) or DSSP led to 4.9% and 6.9% decreases in terms of AUPR, respectively. The results demonstrated that the embeddings learned from the pretrained language model ESM-2 were better than the evolutionary features.

**Table 1.** The predictive performance on the independent test using different features.^a^

Feature group	AUC	AUPR	F1	MCC
EVO	0.717	0.191	0.269	0.185
DSSP	0.680	0.171	0.235	0.142
ESM-2	0.736	0.240	0.291	0.212
EVO + DSSP	0.723	0.200	0.271	0.192
ProtTrans + DSSP	0.750	0.260	0.309	0.214
ESM + DSSP	0.744	0.216	0.293	0.212
ESM-2 + EVO + DSSP	**0.757**	0.254	**0.317**	**0.240**
ESM-2 + DSSP(GraphBepi)	0.751	**0.261**	0.310	0.232

a EVO means the evolutionary features HMM and PSSM.

Bold fonts indicate the best results.

In addition, we investigated the performance of our model when integrating different features. We noted that the performance of EVO could be improved by integrating the structural properties DSSP. Therefore, we also integrated DSSP with ESM-2, which improved by 8.75% in terms of the AUPR metric. These results demonstrated that the structural properties of amino acids such as RSA and secondary structure were sufficient to reserve the complex patterns of epitopes. We then integrated more information including evolutionary features, ESM-2 embeddings, and DSSP.

The results showed that combing all information brought a minor improvement on the independent test set (<0.01 of AUC). These results demonstrated that the ESM-2 model may potentially reserve the evolutionary information of the protein. We found ESM-2 performed slightly better than ProtTrans, likely because they took different network architectures. ESM-2 and ProtTrans outperformed ESM in all metrics, probably because they used more training parameters and datasets. A similar trend could be found on the CV results. It should be noted that the features were selected according to the CV results (Supplementary Table S3 and Note S7).

### 3.3 Comparison with state-of-the-art methods

In this section, we investigated the relative importance of each module in GraphBepi (Table 2) and compared our model with state-of-the-art methods (Table 3). We first compared GraphBepi with two sequence-based methods (Bepipred 2.0 and EpiDope) and four structure-based approaches (ElliPro, Discotope 2.0, epitope3D, and ScanNet; Tubiana et al. 2022) using their default parameters. As ScanNet provided the option to select a transfer learning strategy, we called it ScanNet_T when a transfer learning strategy was used, otherwise ScanNet_WT. As shown in Table 3, our method surpassed the second-ranked method ScanNet_T by 5.5% in terms of AUC, 44.0% in terms of AUPR, 20.4% in terms of F1, and 36.9% in terms of MCC, respectively. We noted that ScanNet_T achieved higher performance than ScanNet_WT, probably because the transfer learning strategy generated profitable parameters for the initial model. The third-ranked method was Discotope-2.0, which achieved comparable performance with method ElliPro in terms of AUC value. The AUPR value of Discotope-2.0 was 3.2% higher than that of ElliPro. However, both of them performed lower than ScanNet_WT, likely because ScanNet_WT applied the deep learning network architecture and transfer learning strategy. Interestingly, although epitope3D was a structure-based method, it performed lower than the sequence-based method EpiDope. It is likely that the experimental structures brought noises due to the flexible characteristic of protein structure. Note that GraphBepi did not have the highest recall since it was an unbalanced measure strongly relying on thresholds. All methods have similar inference time if not considering the time to predict structure through Alphafold2 (Supplementary Note S8).

**Table 2.** The predictive performance of GraphBepi on the independent test when removing each module.^a^

Module	AUC	AUPR	F1	MCC
GraphBepi w/o EGNN	0.706	0.209	0.275	0.194
GraphBepi w/o BiLSTM	0.742	0.239	0.300	0.226
GraphBepi	**0.751**	**0.261**	**0.310**	**0.232**

a w/o represents without the corresponding module.

**Table 3.** Performance comparison of GraphBepi with state-of-the-art methods on the independent test data.^a^

Method	AUC	AUPR	F1	MCC	Rec	Pre
EpiDope	0.547	0.102	0.173	0.046	**0.855**	0.096
Bepipred-2.0	0.648	0.132	0.220	0.126	0.562	0.137
ElliPro	0.632	0.122	0.217	0.123	0.592	0.133
Discotope-2.0	0.655	0.154	0.231	0.136	0.405	0.162
epitope3D	0.577	0.105	0.135	0.039	0.176	0.109
ScanNet_WT	0.648	0.135	0.218	0.121	0.532	0.137
ScanNet_T	0.712	0.182	0.257	0.170	0.440	0.182
GraphBepi	**0.751**	**0.261**	**0.310**	**0.232**	0.393	**0.255**

a ScanNet_T means that method ScanNet uses the transfer learning strategy, while ScanNet_WT means that it does not.

To further investigate the advantages of our method, we analysed the relative importance of each module in GraphBepi by conducting the model ablation study on the test data. As shown in Table 2, the removal of the module BiLSTM caused a decrease of 0.9% and 2.2% in terms of AUC and AUPR. This change indicated that the BiLSTM could capture long-range dependencies of amino acid residues. We also replaced GraphBepi’s BiLSTM with Transformer, resulting in 0.6% and 3% decreases of AUPR for the CV and test results, respectively (Supplementary Table S4). The removal of the EGNN caused the greatest decrease of 4.5% and 5.2% in terms of AUC and AUPR. The results demonstrated that the spatial information was efficiently captured by the EGNN module. In summary, the cooperation of each module achieved the best performance.

### 3.4 Impact of the quality of predicted protein structure

Since our method used predicted structures, it is interesting to investigate the influence of the quality of predicted protein structure models on the downstream BCEs prediction. Here, we evaluated the performance of our model by using the native protein structures and the predicted protein structures from AlphaFold2 and ESMFold. As shown in Table 4, when using native structures or predicted structures from AlphaFold2, our method obtained comparable results. Concretely, the AUC and AUPR to use native structures were only 0.8% and 0.2% higher than the one to use predicted structures from AlphaFold2. Relatively, the performance using predicted structures from AlphaFold2 was slightly higher than the one using ESM-Fold, likely because of the use of multiple-sequence alignment by Alphafold2. On the other hand, when only using the protein sequence, the performance was significantly lower, demonstrating the importance of structural information for BCEs prediction.

**Table 4.** The predictive performance of GraphBepi when using only sequence, or different predicted structures.

Structural information	AUC	AUPR	F1	MCC
Sequence only	0.698	0.204	0.271	0.187
ESM-fold predicted structures	0.746	0.218	0.281	0.208
AlphaFold2 predicted structures	0.751	0.261	0.310	0.232
Native structures	**0.759**	**0.263**	**0.320**	**0.249**

**Figure 3.**
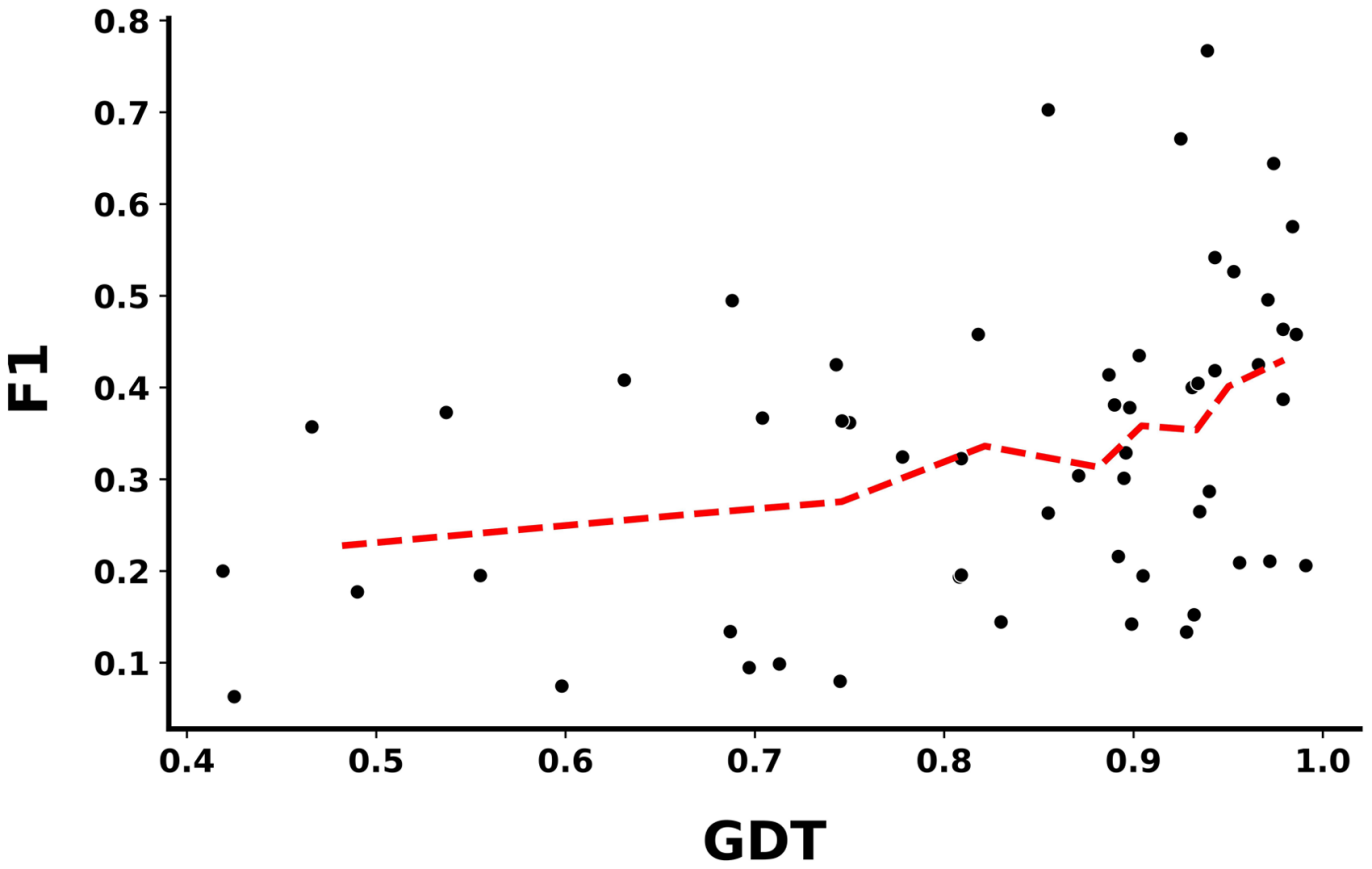
Positive correlation between the AlphaFold2 predicted structure quality evaluated by GDT and the GraphBepi performance evaluated by F1 on the independent test set. The black scatter represents the F1 and GDT score for each antigen. The red line represents the average F1 and GDT for each bin after ranking all antigens by GDT and splitting them into nine bins.

To further investigate the advantages of geometric deep learning employed in our method, we computed the average global distance test (Zemla 2003) (called GDT-score) between the predicted structures and the native structures through the tool Spalign (Yang et al. 2012). Figure 3 shows the quality of the predicted protein structures and the F1 values of each antigen on the independent test data for GraphBepi (black scatters). Specifically, we sorted the antigens based on GDT-score and then split them into nine bins evenly to calculate the mean GDT score and F1 value for every bin (red line). The results showed a positive correlation between the GDT of AlphaFold2 predicting structure and the F1 for its prediction by GraphBepi on the independent test set. The top 20% of antigens with the highest GDT (mean GDT = 0.974) had a mean F1 of 0.406. Whereas, the bottom 20% of antigens with the lowest GDT (mean GDT = 0.563) had a mean average F1 of 0.241. Similar trends could be found in terms of other metrics as shown in Supplementary Fig. S2. These results suggested the importance of the quality of antigens structures predicted by AlphaFold2 for BCEs prediction.

### 3.5 Case study

**Figure 4.**
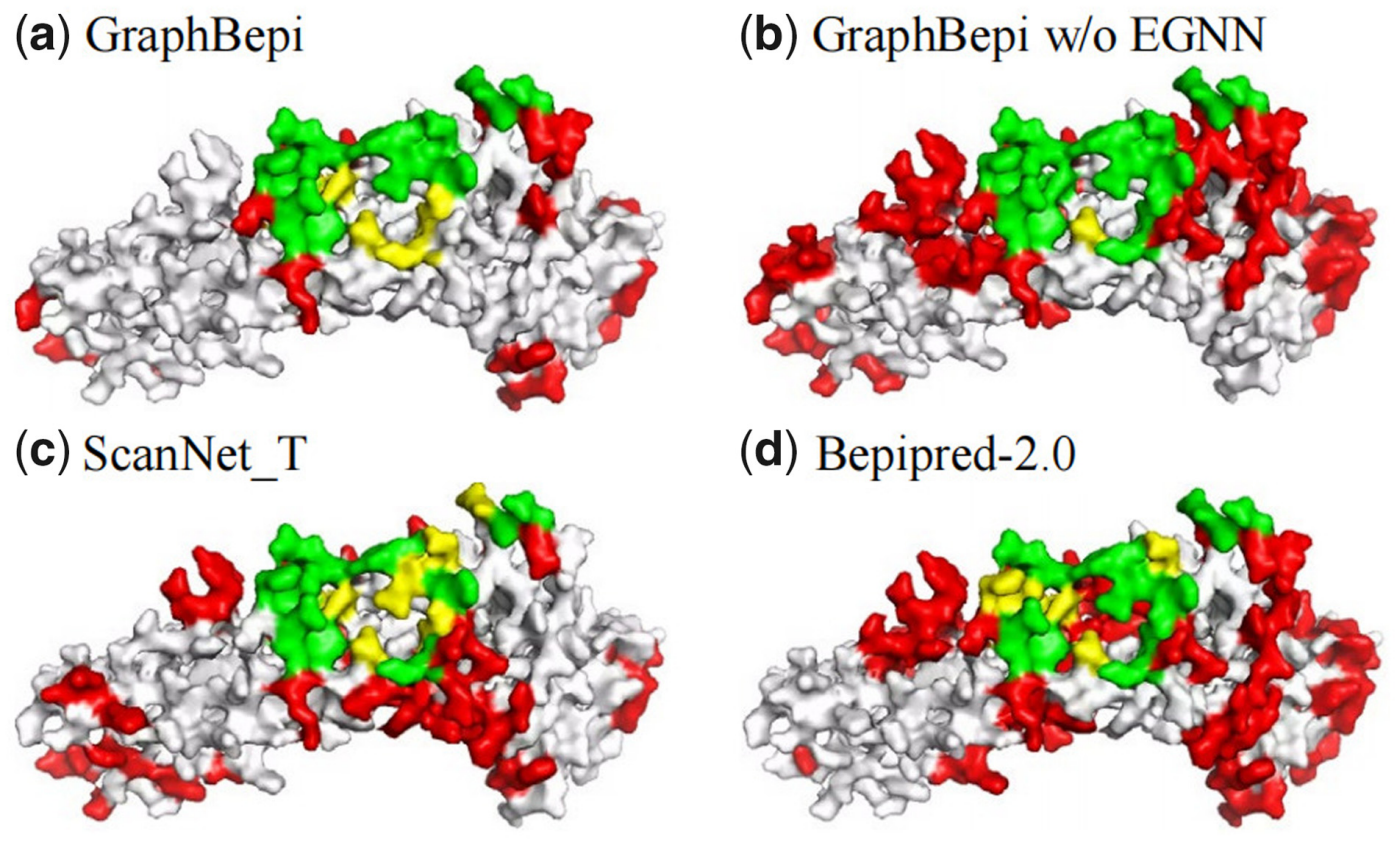
Visualization of a case of the test data (PDB ID: 7S2R, chain A) predicted by GraphBepi (a), GraphBepi without EGNN (b), ScanNet_T (c), and Bepipred-2.0 (d). True positives, false negatives, and false positives are colored in green, yellow, and red, respectively.

To demonstrate the superiority of GraphBepi, we visualized an example (PDB ID: 7S2R, chain A) from the independent test. Figure 4 shows the BCEs predictive results of GraphBepi, GraphBepi without EGNN, the second-ranked method ScanNet_T, and the sequenced-based method Bepipred-2.0. Among the 17 epitopes out of 197 residues, GraphBepi predicted 37 binding residues, of which 13 were TP, resulting in an AUPR of 0.558, F1 of 0.481, and MCC of 0.454. By comparison, the GraphBepi without EGNN predicted 93 binding residues, of which 16 were TP, resulting in a lower AUPR of 0.451, F1 of 0.291, and MCC of 0.289. The results showed that the spatial information captured by the EGNN module could help our method accurately identify the epitopes and reduce the false positive rate. By comparison, the structure-based method ScanNet_T predicted 57 binding residues, of which 11 were TP, resulting in an AUPR of 0.332, F1 of 0.297, and MCC of 0.242. The sequenced-based Bepipred-2.0 predicted 87 binding residues, of which 12 were TP, resulting in a lower AUPR of 0.127, F1 of 0.226, and MCC of 0.157. We also included the predictions for other competing methods in Supplementary Fig. S3.

## 4 Discussion

Identifying BCEs is an essential step for guiding rational vaccine development and immunotherapies. Here, we propose GraphBepi, a novel graph-based model, for accurate epitope prediction by using structural information predicted from AlphaFold2. GraphBepi applies the EGNN to capture the predicted protein structures, and leverages the BiLSTM to capture long-range dependencies from sequences. The low-dimensional representations learned from EGNN and BiLSTM are then combined to predict BCEs. Through comprehensive tests on the curated epitope dataset, GraphBepi was shown to outperform the state-of-the-art methods.

Although several sequence-based methods have also been designed for identifying the BCEs such as EpiDope and Bepipred 2.0, they obtain limited performance since they only use the contextual features of the sequential neighbors. By comparison, structure-based methods try to solve these problems by considering spatial information, but they are not applicable to most proteins due to a lack of known tertiary structures. At the same time, these methods often use evolutionary information that is time-consuming. Here, we employed AlphaFold2 predicting structures to capture spatial information and ESM-2 to effectively represent protein sequence information. The comprehensive tests indicated that our model obtains superior performance compared to the state-of-the-art tools.

In spite of the advantages, GraphBepi can be enhanced in several aspects. First, the performance of our model is influenced by the quality of antigen structures predicted from AlphaFold2. This might be relieved by adding other sequence-derived features to increase the robustness of the model. We will explore these challenges in future work. Second, the employed EGNN is a blackbox model. The future combination with explainable models might interpret the prediction or even improve the performance (Rao et al. 2022).

In summary, we have demonstrated that GraphBepi provides a novel graph-based model for accurately predicting BCEs. We also provide the web server of GraphBepi at http://bio-web1.nscc-gz.cn/app/graphbepi.

## Supplementary Material

btad187_Supplementary_Data
