# Supplementary material for: Identifying B-cell epitopes using AlphaFold2 predicted structures and pretrained language model
Source: Bioinformatics. 2023 Apr 11;39(4):btad187. doi: 10.1093/bioinformatics/btad187 (PMC10126322; doi:10.1093/bioinformatics/btad187)
Supplement: btad187_Supplementary_Data [file btad187_supplementary_data.docx]

**Supplementary Information**

Identifying B-cell epitopes using AlphaFold2 predicted structures and pretrained language model

Yuansong Zeng^1,#^, Zhuoyi Wei^1,#^, Qianmu Yuan^1^, Sheng Chen^1^, Weijiang Yu^1^, Yutong Lu^1^, Jianzhao Gao^3,*^, and Yuedong Yang^1,2*^

^1^School of Computer Science and Engineering, Sun Yat-sen University, Guangzhou 510000, China.

^2^Key Laboratory of Machine Intelligence and Advanced Computing (MOE), Guangzhou 510000, China.

^3^School of Mathematical Sciences and LPMC, Nankai University, Tianjin, China.

*To whom correspondence should be addressed.

# These authors contributed equally to this work.

**Table S1.** The predictive performance of GraphBepi on the CV and the independent test.

| Method | Category | Rec | Pre | F1 | MCC | AUC | AUPR |
| --- | --- | --- | --- | --- | --- | --- | --- |
| GraphBepi | CV | 0.502 | 0.235 | 0.320 | 0.212 | 0.723 | 0.245 |
|  | Test | 0.393 | 0.255 | 0.310 | 0.232 | 0.751 | 0.261 |

**Table S2.** Performance comparison with the geometric-agnostic baseline model transformer on the independent test.

| Dataset | Method | Rec | Pre | F1 | MCC | AUC | AUPR |
| --- | --- | --- | --- | --- | --- | --- | --- |
| Independent  test data | transformer | 0.514 | 0.173 | 0.259 | 0.176 | 0.705 | 0.197 |
|  | GraphBepi | 0.393 | 0.255 | 0.310 | 0.232 | 0.751 | 0.261 |
|  | GraphBepi(GCN) | 0.528 | 0.206 | 0.296 | 0.222 | 0.746 | 0.232 |

*Note*: GraphBepi(GCN) indicates that the EGNN module of the GraphBepi is replaced by the classic GCN module without considering edge features.


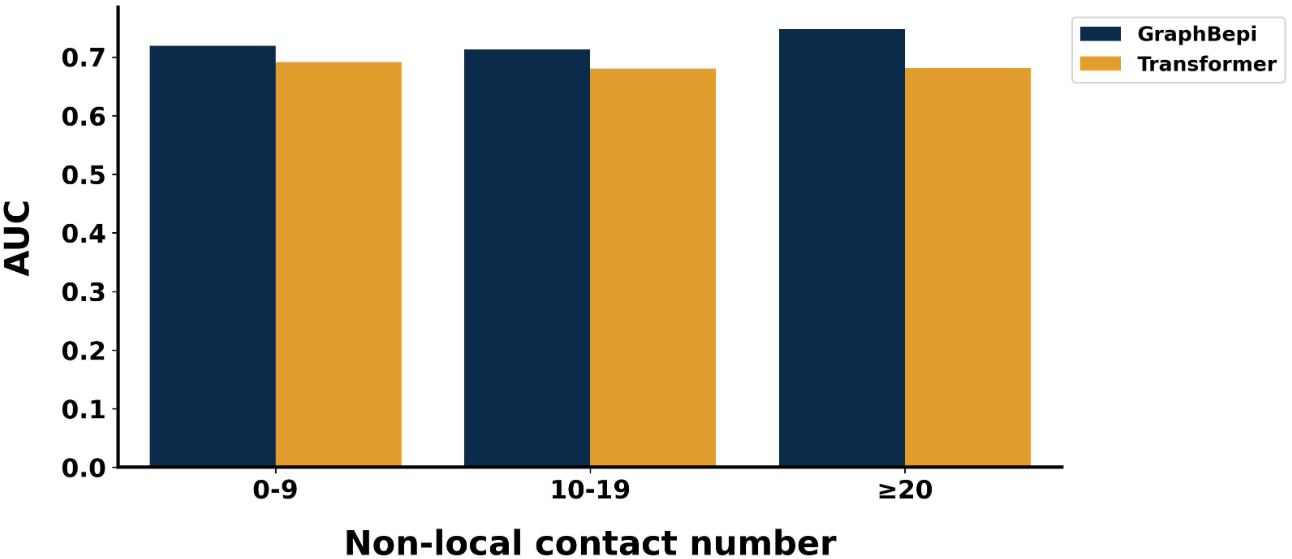


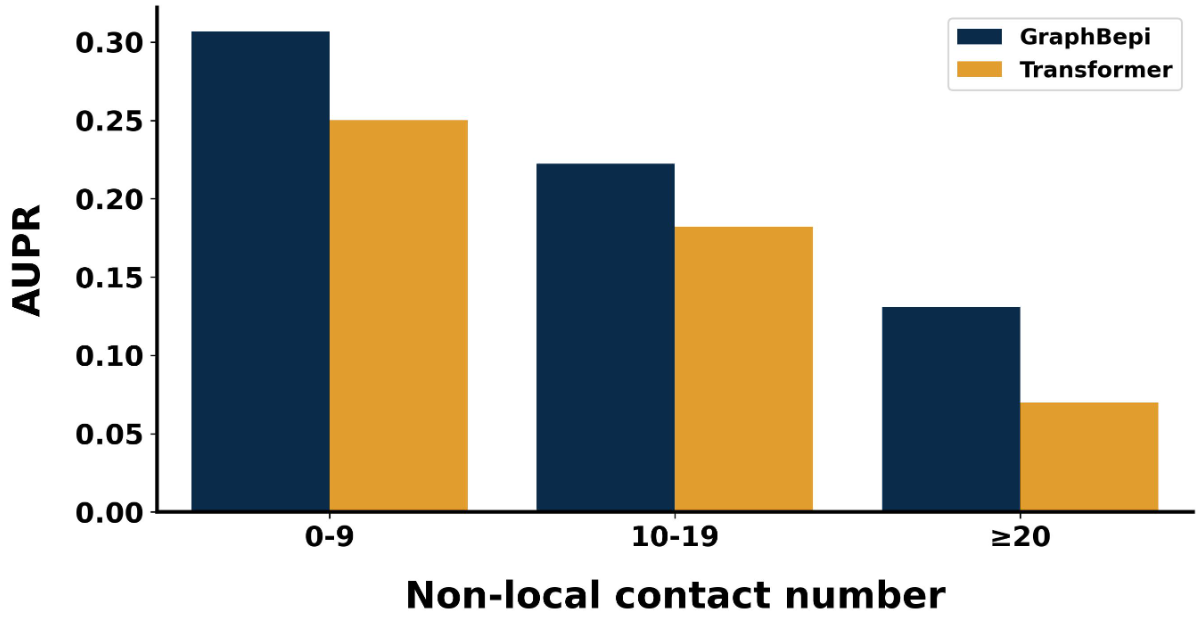


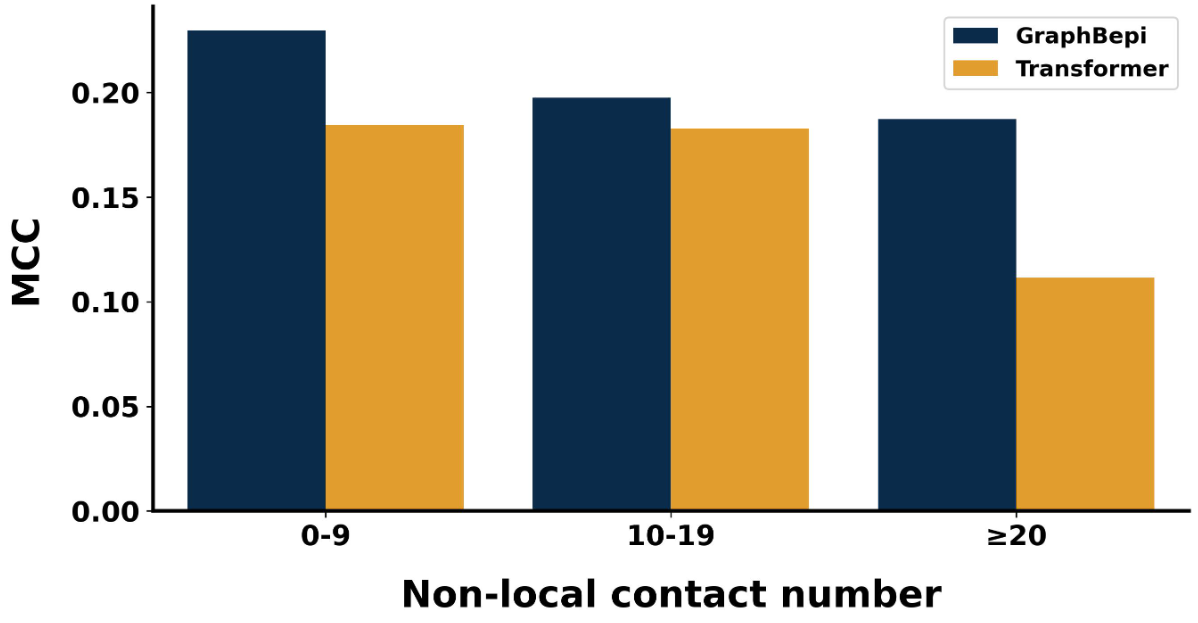


**Figure S1.** The AUC, AUPR, MCC of GraphBepi and transformer on amino acids with different numbers of non-local contacts in the independent test.


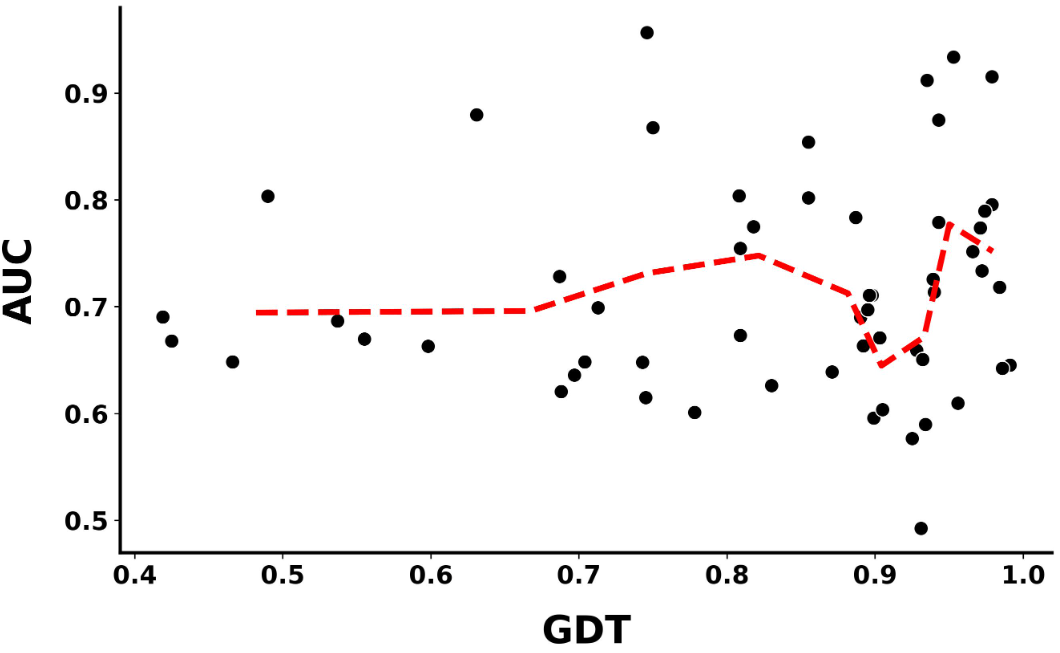


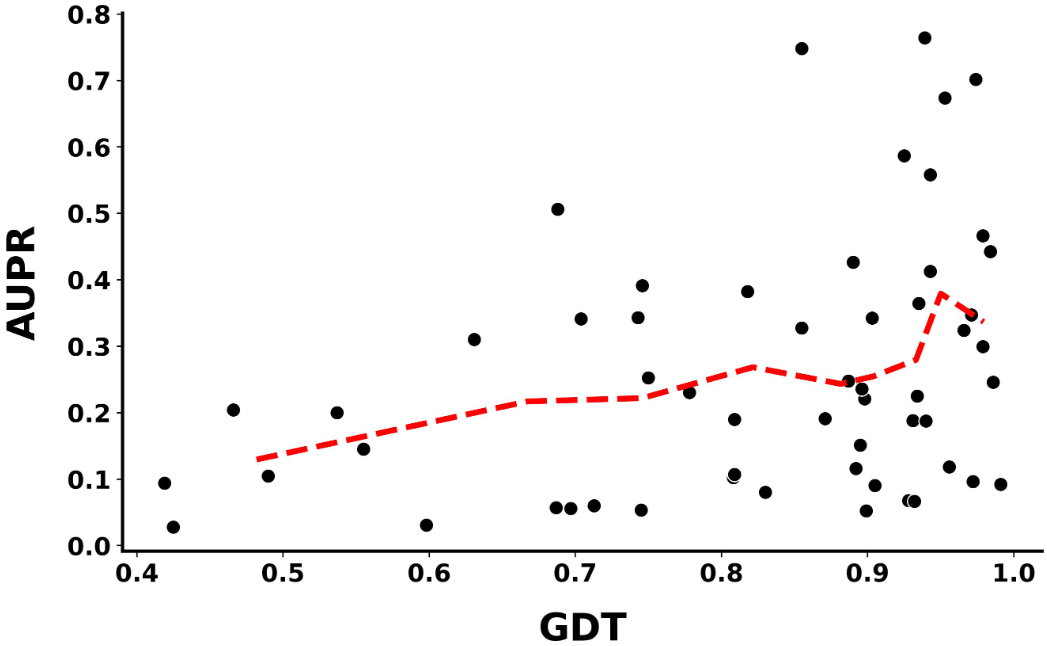


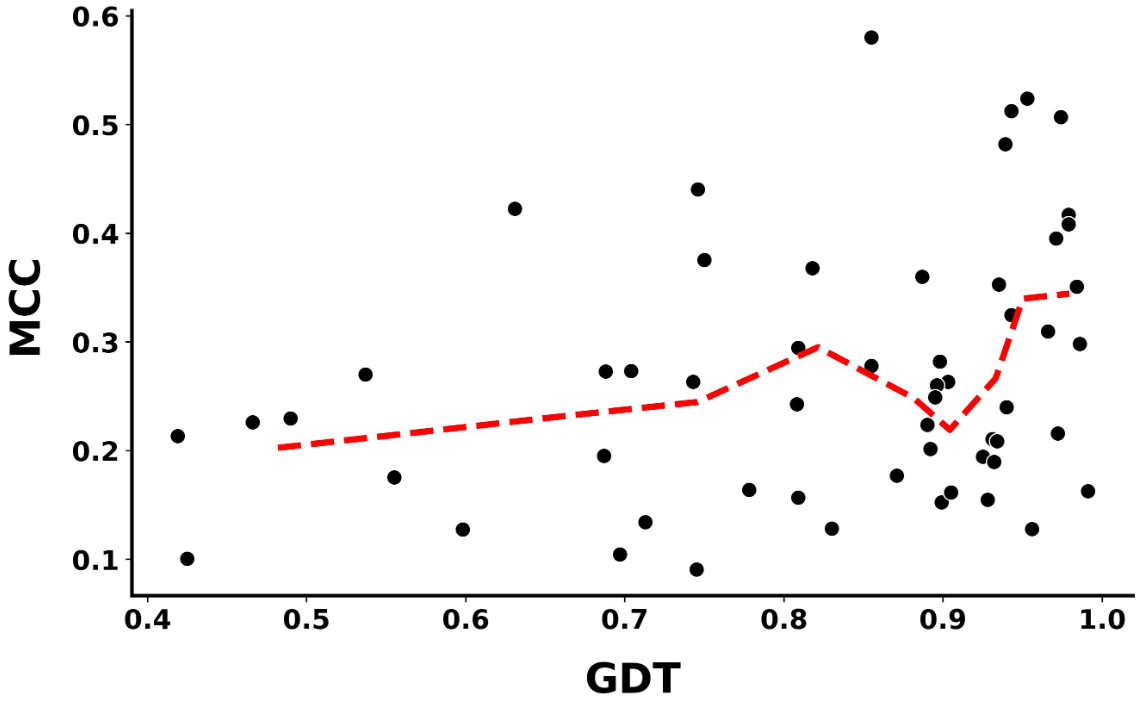


**Figure S2.** Positive correlation between the AlphaFold2 predicted quality measured by GDT and the GraphBepi performance measured by AUC, AUPR, and MCC on the independent test set. The black scatter indicates the GDT and AUC for each protein, while the red line indicates the average GDT and AUC for each bin after sorting all antigens by GDT and dividing them into nine bins.


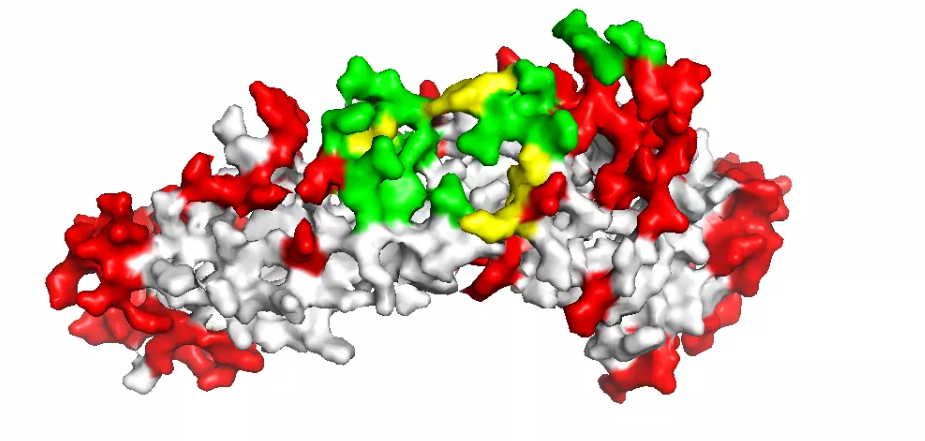


1. ScanNet_WT


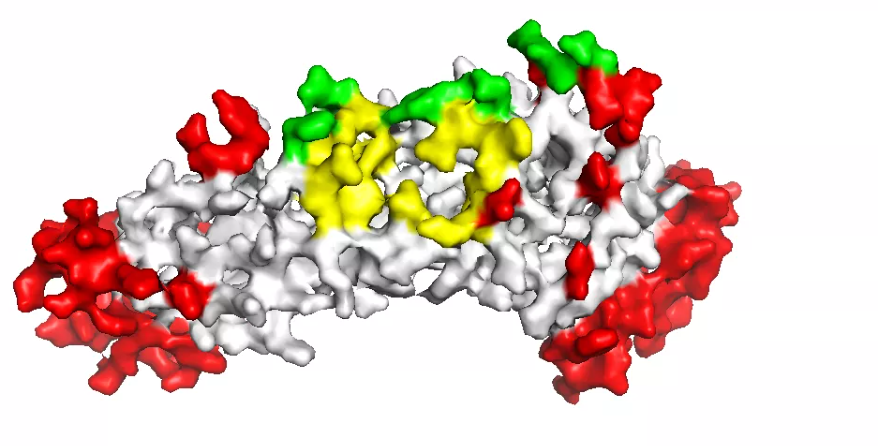


1. ElliPro


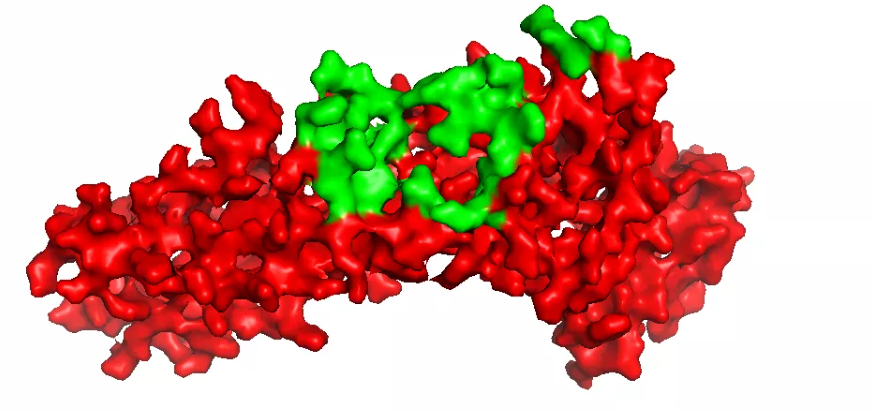


1. EpiDope


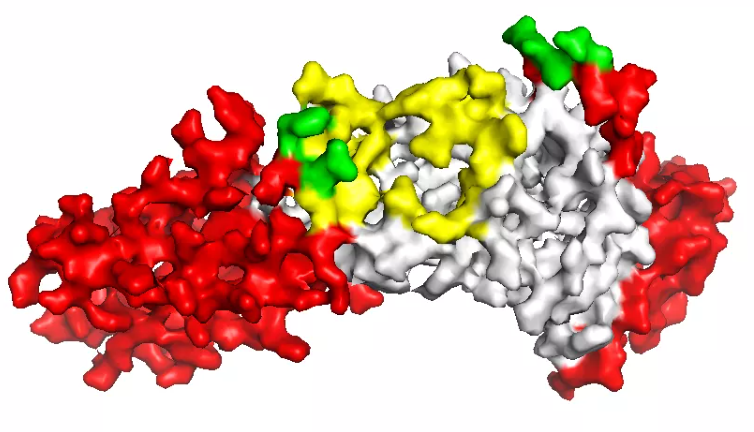


1. Discotope-2.0

**Figure S3.** Visualization of an example (PDB ID: 7S2R, chain A) from the test data predicted by (A) ScanNet_WT, (B) ElliPro, (C) EpiDope, and (D) Discotope-2.0. True positives, false negatives, and false positives are colored in green, yellow, and red, respectively.

**Supplementary Note 1**

The ESM-2 language model was a pretrained language model that was trained to predict randomly selected amino acids in a protein sequence from their context in the rest of the sequence. ESM-2 is a new family of transformer protein language models, which scales from 8 million parameters up to 15 billion parameters. For each given residue, the last layer of the encoder of ESM-2 is a 2560-dimensional vector containing evolutional information because ESM-2 was trained on unprecedented large datasets of protein sequences and structures. The vector has been shown able to accurately predict secondary structure and contact maps. Thus, the feature vector of each residue may be beneficial to identify B-cell epitopes.

**Supplementary Note 2**

*PSSM:* The position-specific scoring matrix (PSSM) is generated by applying the tool PSI-BLAST to seek the candidate sequence against UniRef90 using three iterations and an E-value of 0.001. The size of the produced position-specific scoring matrix (PSSM) is L×20. Each amino acid is encoded as a vector with 20 elements, which represents the probability that 20 amino acids will occur at that position.

*HMM:* The HMM profile was generated by searching the query sequence against UniClust30 through HHblits with default parameters. The HMM profile was represented as the HMM matrix with the dimension of L×30. Concretely, the HMM matrix is composed of 20 columns of observed frequencies for 20 amino acids in homologous sequences, 7 columns of conversion frequencies, and 3 columns of local diversity. The integer in the HMM is equal to the negative logarithm of the frequency of the amino acid multiplied by 1000. In this study, only the first 20 columns were used, as in the reference (Yuan, et al., 2022).

**Supplementary Note 3**

AlphaFold2 is a deep learning-based system to predict the 3D structure of proteins from their amino acid sequences. AlphaFold2 has the potential to revolutionize the field of protein structure prediction and could lead to a better understanding of how proteins work and how they interact with each other. AlphaFold2 has integrated physical and biological knowledge about protein structure, information of multi-sequence alignment (MSA) and the sophisticated design of the deep learning algorithm. In the 14th Critical Assessment of Protein Structure Prediction, AlphaFold2 has shown the ability in predicting the structure of the protein with atomic accuracy and demonstrated accuracy competitive with experiments on a large number of cases. In particular, the trunk of AlphaFold2 processes the inputs through repeated layers of a novel neural network block, which we call the Evoformer, to produce the representation. The Evoformer blocks contain several attention-based and non-attention-based components. The trunk of AlphaFold2 is followed by the structure module, which introduces a 3D structure for each residue in the protein.

**Supplementary Note 4**

**The Bidirectional LSTM module**

The Long Short-Term Memory (LSTM) (Hochreiter and Schmidhuber, 1997) is a classic algorithm for capturing long-range dependencies, which is widely used in protein sequence encoding. The bidirectional LSTM (BiLSTM) model (Graves and Schmidhuber, 2005) adds one more LSTM layer and reverses the direction of information. Briefly, it means that the input sequence flows backward in the additional LSTM layer. In this study, we use a bidirectional LSTM to process antigen sequences since they don’t have a specific direction. Concretely, LSTM cell is a particular RNN (recurrent neural network) cell (Schuster and Paliwal, 1997), which will be computed by the forget gate, input gate, and output gate. The LSTM first uses the forget gate to decide what information needs to be discarded in the LSTM cell through $h_{t-1}{\left( previous output \right)\mathrm{and} x}_{t}$ (the input of current time step) as follows:

$f_{t}=\sigma\left( W_{f}\left[ h_{t-1},x_{t} \right] +b_{f} \right)$ (1)

where [.] and $\sigma$ represent the concatenation operation and the sigmoid function, respectively. $W_{f}$ and $b_{f}$ are the learnable parameters.

The input gate is applied to decide what information needs to be stored in the LSTM cell. Specifically, the input gate employs activation functions of sigmoid$\sigma$ and tanh to determine what values we will update $(i_{t})$ and generate the term $j_{t}$ that will be added to the state:

$i_{t}=\sigma\left( W_{i}\left[ h_{t-1},x_{t} \right]+b_{i} \right)$ (2)

$j_{t}=tanh(W_{j}\left[ h_{t-1},x_{t} \right]+b_{j})$ (3)

We then update the old state of LSTM cell $C_{t-1}$ as follows:

$C_{t}=f_{t}C_{t-1}+i_{t}j_{t}$ (4)

At last, the output gate leverages a sigmoid function $\sigma$ to determine what part of $C_{t}$ will be output $\left( Q_{t} \right)$ as follows:

$Q_{t}=\sigma(W_{Q}\left[ h_{t-1},x_{t} \right]+b_{Q})$ (5)

$h_{t}=Q_{t}tanh(C_{t})$ (6)

In this study, we use a bidirectional LSTM (BiLSTM) to process antigen sequences since they don’t have a specific direction

${}_{h_{t}}^{\to}={}_{LSTM}^{\to}{(x_{t}, {}_{h_{t+1}}^{\to})}$ (7)

${}_{h_{t}}^{\leftarrow}={}_{LSTM}^{\leftarrow}{(x_{t}, {}_{h_{t+1}}^{\leftarrow})}$ (8)

where ${}_{h_{t}}^{\to}{and} {}_{h_{t}}^{\leftarrow}{}$ will be combined by the concatenation operation at the time step t as follows:

$h_{t}=[{}_{h_{t}}^{\to}, {}_{h_{t}}^{\leftarrow}{}]$ (9)

$H=[h_{0}, \ldots h_{L}]$ (10)

We denote the output of the BiLSTM layer as H for simplicity. In this study, the BiLSTM is employed for learning the DSSP structural properties and the sequence embeddings obtained from ESM-2, respectively.

**Supplementary Note 5**

These three edge types represented different edge relationships. Sequential edges took account of the sequential distances of two residues in the sequential space. Radius edges calculated the Euclidean distance between two residues using their spatial coordinates. Since the scales of spatial coordinates may vary among different antigens, a node would be also connected to its k-nearest neighbors (KNN edges) based on the Euclidean distance.

We followed the reference (Zhang, et al., 2022) to construct six edge features, resulting in a total of 51 dimensions. Specifically, we used the one-hot encoding of residue types with an additional dimension for unknown types as edge node features, denoted as $f\in\left\{ 0,1 \right\}^{(n\times21)}$. The dimensions of the sequential edge, radius edge, and KNN edge were 5, 1, and 1, respectively (see the section Graph construction). The total dimensions of the sequential and spatial distances between two connected residues were 2.

**Supplementary Note 6**

***Implementation details:*** Our method conducted 10-fold cross-validation (CV) for the training dataset that was split into 10-folds randomly. Each time, our method was trained on 9-folds and then tested on the one rest fold. We performed this process ten times. The average performance of our method on the 10-folds was treated as the final validation performance. For the independent test dataset, all ten trained models in the CV were used for making predictions, which were then averaged as the final testing results. In addition, the number of layers and hidden units of BiLSTM were set to 3 and 128, respectively. For the EGNN module, the number of layers and hidden dimensions were set to 2 and 256, respectively. We leveraged the Adam optimizer with a batch size of 4 and a learning rate of 1e-6 for model optimization on the binary cross-entropy loss. Our method was implemented through Pytorch and python. We fixed the training epoch to 300 epochs since the average performance of the validated data for the 10 trained models was the highest at this epoch.

We scanned the hyperparameters of the learning rate [1e-4, 1e-5, 1e-6, 1e-7], the batch size [2, 4, 6, 8], the number of layers of BiLSTM [1, 2, 3, 4, 5], the hidden dimensions of BiLSTM [32, 64, 128, 256], the number of layers of EGNN [2, 4, 6], the hidden dimensions of EGNN [32, 64, 128, 256], the number of layers of multilayer perceptron [1, 2, 3, 4], the hidden dimensions of multilayer perceptron [128, 256, 512, 1024] through the grid search, and then selected the optimal hyperparameters based on AUPR (results not shown).

**Supplementary Note 7**

The features were selected according to the CV, not the test results. We added the CV results in Supplementary Table S3. We didn’t include HMM and PSSM features (Evolutionary features) because they were time-consuming, costing ~10 minutes for a 200-residue protein. In addition, the use of Evolutionary features only achieved essentially the same performance as GraphBepi on the CV and test results (AUC values of 0.729 and 0.723 on the CV results; AUC values of 0.757 and 0.751 on the test results).

**Table S3.** The predictive performance on the CV data using different features.

| Feature group | AUC | AUPR | F1 | MCC | Rec | Pre |
| --- | --- | --- | --- | --- | --- | --- |
| Evo | 0.701 | 0.221 | 0.299 | 0.186 | 0.568 | 0.203 |
| DSSP | 0.687 | 0.207 | 0.289 | 0.172 | **0.570** | 0.194 |
| ESM-2 | 0.720 | 0.239 | 0.322 | 0.214 | 0.521 | 0.233 |
| Evo +DSSP | 0.708 | 0.232 | 0.304 | 0.190 | 0.511 | 0.216 |
| ProtTrans + DSSP | 0.722 | 0.245 | 0.319 | 0.212 | 0.503 | 0.234 |
| ESM+ DSSP | 0.720 | 0.244 | 0.322 | 0.214 | 0.528 | 0.232 |
| ESM-2 + EVO + DSSP | **0.729** | 0.248 | **0.325** | **0.219** | 0.553 | 0.230 |
| ESM-2 + DSSP(GraphBepi) | 0.723 | 0.245 | 0.320 | 0.212 | 0.502 | **0.235** |

**Supplementary Note 8**

To run a protein of 200 residues on an Nvidia GeForce RTX 3090 GPU, GraphBepi required about one second. In comparison, ESM-2 required 50 seconds and Alphafold2 required about 30 minutes. Thus, we included two models on our webpage with Alphafold2 structures or ESM-fold structures. The GraphBepi using ESM-fold was in the same level of speed as other sequence-based methods (such as EpiDope or Bepipred-2.0, within 2 minutes). The other structure-based methods (such as ScanNet, Discotope-2.0, and ElliPro) can also be done within 2 minutes, while epitope3D took about 3 minutes.

Table S4. The performance of GraphBepi when applying Transformer or BiLSTM on the CV and test data.

|  | CV | | | | Test | | | |
| --- | --- | --- | --- | --- | --- | --- | --- | --- |
| Model | AUC | AUPR | F1 | MCC | AUC | AUPR | F1 | MCC |
| GraphBepi (Transformer) | 0.715 | 0.239 | 0.316 | 0.206 | 0.740 | 0.231 | 0.304 | 0.226 |
| GraphEPI (BiLSTM) | **0.723** | **0.245** | **0.320** | **0.212** | **0.751** | **0.261** | **0.310** | **0.232** |

References

Graves, A. and Schmidhuber, J. Framewise phoneme classification with bidirectional LSTM and other neural network architectures. *Neural networks* 2005;18(5-6):602-610.

Hochreiter, S. and Schmidhuber, J. Long short-term memory. *Neural computation* 1997;9(8):1735-1780.

Schuster, M. and Paliwal, K.K. Bidirectional recurrent neural networks. *IEEE transactions on Signal Processing* 1997;45(11):2673-2681.

Yuan, Q.*, et al.* AlphaFold2-aware protein–DNA binding site prediction using graph transformer. *Briefings in Bioinformatics* 2022;23(2):bbab564.

Zhang, Z.*, et al.* Protein representation learning by geometric structure pretraining. *arXiv preprint arXiv:2203.06125* 2022.
